# Inhibition of metastatic brain cancer in Sonic Hedgehog medulloblastoma using caged nitric oxide albumin nanoparticles

**DOI:** 10.3389/fonc.2023.1129533

**Published:** 2023-05-03

**Authors:** Bohdan J. Soltys, Katie B. Grausam, Shanta M. Messerli, Carleton J. C. Hsia, Haotian Zhao

**Affiliations:** ^1^ AntiRadical Therapeutics LLC, Sioux Falls, SD, United States; ^2^ Cancer Biology and Immunotherapies, Sanford Research, Sioux Falls, SD, United States; ^3^ Department of Pediatrics, University of South Dakota, Vermillion, SD, United States; ^4^ Department of Biomedical Sciences, New York Institute of Technology, Old Westbury, NY, United States

**Keywords:** Sonic Hedgehog, medulloblastoma, metastasis, leptomeningeal dissemination, hematogenous dissemination, nitroxide, reactive oxygen species, reactive nitrogen species

## Abstract

Medulloblastoma is a tumor of the cerebellum that metastasizes to the leptomeninges of the central nervous system (CNS), including to forebrain and to spinal cord. The inhibitory effect of polynitroxylated albumin (PNA), a caged nitroxide nanoparticle, on leptomeningeal dissemination and metastatic tumor growth was studied in a Sonic Hedgehog transgenic mouse model. PNA treated mice showed an increased lifespan with a mean survival of 95 days (n = 6, *P*<0.05) compared with 71 days in controls. In primary tumors, proliferation was significantly reduced and differentiation was significantly increased (*P*<0.001) as shown by Ki-67^+^ and NeuN^+^ immunohistochemistry, while cells in spinal cord tumors appeared unaffected. Yet, histochemical analysis of metastatic tumor in spinal cord showed that the mean total number of cells in spinal cord was significantly reduced in mice treated with PNA compared to albumin vehicle (*P*<0.05). Examination of various levels of the spinal cord showed that PNA treated mice had significantly reduced metastatic cell density in the thoracic, lumbar and sacral spinal cord levels (*P*<0.05), while cell density in the cervical region was not significantly changed. The mechanism by which PNA may exert these effects on CNS tumors is discussed.

## Introduction

Cancerous brain tumors such as medulloblastoma (MB) and glioblastoma begin as primary tumors with the brain and metastasize to secondary locations within the central nervous system (CNS) and possibly elsewhere. Owing to the blood brain barrier (BBB), pharmacological treatment of brain tumors is problematic due to lack of drug entry into the CNS (1, 2). Successful therapeutic intervention for malignant tumors could in principle be accomplished at primary tumors, on tumor cells in transit to secondary sites and/or at sites of secondary tumor formation. In the case of solid tumors that originate outside the brain the spread of metastatic tumor cells occurs primarily through the bloodstream (hematogenous dissemination) and in certain cases through the lymphatic system (3). In MB, the most common malignant pediatric brain cancer, primary tumors originate within the cerebellum and hindbrain, and metastasize to leptomeningeal membranes of the forebrain and spinal cord (4–7). Despite treatments, ~30% of patients with MB succumb, while survivors suffer from long-term side-effects due to treatments (4–7). Until recently, it was assumed that metastatic spread in MB occurred only through the cerebrospinal fluid (CSF). There is now evidence that MB may also spread by means of hematogenous dissemination (8, 9). This finding lays open the possibility that metastasis could in principle be inhibited by drugs that act within the vasculature without crossing the BBB.

Nanoparticles (NPs) are at the forefront in the development of anti-cancer treatments (10, 11). First generation NPs are exemplified by Abraxane, which has had broad clinical success and next generation NPs are being developed (11, 12). PolyNitroxylated Albumin (PNA) belongs to a class of caged nitric oxide NPs (CNO-NP) drugs developed by Dr. Carleton Hsia and associates. Another example is PolyNitroxylatedPEGylated Hemoglobin (PNPH). The actions of CNO-NPs is based firstly on the cyclic nitroxide moieties that are covalently linked to the macromolecule comprising the NP. The macromolecules themselves and any further chemical modifications may have ancillary activities. Low-molecular-weight nitroxides by themselves have a long history of studies showing beneficial effects in diverse diseases including cancer (13–15). Cyclic nitroxides can scavenge or dismutate reactive oxygen and reactive nitrogen species (ROS/RNS) which are overproduced in various diseases (16). The effectiveness of low-molecular-weight nitroxides is limited by a large volume of distribution and exchange between intracellular and extracellular spaces, rapid bioreduction and loss of activity, and toxicities. By covalent coupling of nitroxides to a macromolecule, NP-bound nitroxides do not normally cross membranes or enter cells, while the biologic half-life of the nitroxides is extended. The pharmacological action of CNO-NPs is expected to be confined mainly to within the vasculature. Using live animal imaging, PNA as the prototype example of this class of drugs has been directly shown in cancer studies to increase blood flow into peripheral solid tumors *in vivo* within minutes (17, 18). Furthermore, PNA has been shown to significantly increase animal survival and to reduce metastasis when used in combination with standard of care chemotherapeutics in a mouse model of breast cancer, attributable to effects within the vascular compartment (17). To date, both PNA and PNPH have been shown to have pharmacological efficacy in pre-clinical models of a wide variety of major medical conditions (17–38).

We previously developed a Sonic Hedgehog (SHH) MB transgenic murine model in which brain tumors arise spontaneously in the cerebellum at 6 weeks of age and metastasize to the forebrain and to spinal cord (39). SHH belongs to one of four main types of MB as defined by the primary developmental pathway activated: 1) SHH Pathway 2) wingless (WNT) 3) Group 3 4) Group 4 (5, 6, 8, 40). While SHH MB has an intact blood brain barrier (BBB) and apparently normal vasculature, WNT MB has an aberrant vasculature and lacks an intact BBB (40, 41). As a result, WNT MB has been found to be highly drug treatable while SHH MB is not (40, 41). The surprising finding we report is that PNA is highly effective in treating our SHH MB transgenic mouse model.

## Methods

### Transgenic mice and PNA treatment

Transgenic SHH MB mice (MAP mice) were generated as described previously (39). PNA is produced by the reaction of 4-(2-bromoacetamido)-2,2,6,6-tetramethyl-1-piperidinyloxy (BrAcTPO) (Sigma-Aldrich, St. Louis, MO) and human serum albumin, and manufactured as a 10% solution. PNA (Lot 032597, AntiRadical Therapeutics LLC, Sioux Falls, SD) or 25% USP grade human serum albumin (Baxter, Deerfield, IL) vehicle was injected intraperitoneally three times a week (n = 6 for PNA treatment; n = 3 for vehicle control), starting at six weeks of age. The final dosage in all animals was 10 ml kg-1. This dosing was based on that used previously in breast cancer mouse studies (17) and in other systems. The PNA dose administered was near maximum due to injection volume considerations and the size of mice.

### Tissue processing

Animals were sacrificed and perfused at end-point with cold phosphate buffered saline (PBS) followed by cold 4% paraformaldehyde (PFA). Whole brain and spinal column were dissected and fixed in 4% PFA overnight at 4°C and processed for paraffin embedding and sectioning. Tissue sections were deparaffinized with CitriSolv (Decon Labs, King of Prussia, PA) and rehydrated through graded ethanol solutions. For frozen tissues, PFA fixed samples were further equilibrated in 20% sucrose for 24-48 hours at 4°C, embedded in TissueTek-Optimal Cutting Temperature (O.C.T.) compound (Sakura Finetek, Torrance, CA), and stored at -80°C. Frozen tissue blocks were sectioned at 15-20 µm thickness on a cryostat.

### Immunohistochemistry

Immunostaining was carried out as described previously (39). Heat-induced epitope retrieval was performed for paraffin-embedded tissue sections using Rodent Decloaker (RD913 L, Biocare Medical, Concord, CA). Endogenous peroxidase activities were quenched with 3% H_2_O_2_ for 10 minutes. Tissue sections were treated with 10% normal serum in PBS-0.1% Triton X-100 for 1 hour and incubated with primary antibodies for 1 hour at room temperature. After three washes of 5 minutes each with PBS, biotinylated secondary antibodies were applied for 1 hour at room temperature, after which avidin enzyme complex and substrate/chromogen were used for color development (Vector Laboratories, Burlingame, CA). Stained tissue sections were counterstained with hematoxylin and eosin (H&E). If suboptimal staining occurred due to suboptimal fixation or the staining was uninterpretable, the slides were not included in the analysis and sample size was reduced.

### Cell quantitation

For cell proliferation analysis, the number of Ki-67^+^ cells in 300 tumor cells was assessed from five distinct fields of each sample. For cell differentiation analysis, the number of NeuN^+^ cells in 300 tumor cells was assessed from five distinct fields of sample. The percentage of Ki-67^+^ or NeuN^+^ cells in total tumor cell population was calculated by averaging the number of positively-stained cells per 100 tumor cells of all samples for each treatment.

### Tumor cell number and density

To quantify spinal tumors, high magnification images were taken of cross sections at different spinal cord levels. Boxes of 600 µm in perimeter were placed in the tumor region and the number of tumor cells within each box was counted. The area encompassed by tumor mass was calculated to determine total number and density of tumor cells on each section using StereoInvestigator software (MBF Bioscience, Williston, VT). Total tumor cell number and density was determined by averaging results from four representative sections at each spinal cord level for each treated animal. If no tumor cells were found at a certain level, the density was scored as zero to demonstrate the effects of PNA.

### Image acquisition

Light microscopic images were obtained using a Nikon SMZ1000 Stereomicroscope or a Nikon Eclipse 90i microscope system (Nikon Instruments, Melville, NY).

### Statistics

The technician performing cell scoring and quantitation was blinded to the treatment condition. Other investigators were not blinded during experiments and outcome assessment. Statistical analyses were performed with GraphPad Prism 6.0 (GraphPad Software Inc., La Jolla, CA). All pooled data were expressed as the mean ± standard error of the mean (SEM). Differences between groups were compared using an unpaired two-tailed t test. Results were considered significant at *P < 0.05; **P <0.01; ***P <0.001; ****P <0.0001. The significance of intergroup differences in the incidence of primary tumor, leptomeningeal disease, and metastasis was assessed using Fisher’s exact test.

### Animal welfare

Animals were housed in the AAALAC-accredited Laboratory Animal Research Facility at Sanford Research in accordance with NIH guidelines. All animal experimental procedures were approved by Sanford Research Institutional Animal Care and Use Committee (IACUC) and performed following national regulatory standards.

## Results

### Leptomeningeal dissemination and brain metastasis

SHH MB mice used in this study were generated as previously reported and are referred to as MAP mice (39). MAP mice were treated with PNA or albumin vehicle starting at six weeks of age, a time point at which primary tumors in cerebellum are present and small clumps of tumor cells are present in CSF, indicative of leptomeningeal disease (39). Animals received 3 treatments per week until endpoint (end of life). At endpoint, all vehicle control mice showed leptomeningeal dissemination and metastasis to both the forebrain and to spinal cord. In PNA treated mice only 1 of 6 mice exhibited leptomeningeal dissemination and metastasis to the forebrain and 4 of 6 exhibited metastasis to the spinal cord (**Table 1**, *P*<0.05). Thus, PNA prevented metastasis to the forebrain in ~83% of animals and prevented metastasis to the spinal cord in ~33% of animals.

**Table 1:** PNA treatment and incidence of primary tumors in cerebellum, leptomeningeal disease (LMD), brain metastasis to forebrain and spinal cord metastasis.

Treatment	Tumor (#)incidence	LMD (#)incidence	Brain Met (#)incidence	S.C. Met (#)incidence	Average Age (days)
*Polynitroxyl Albumin*	100%(6/6)	17%(1/6)*	17%(1/6)*	67% (4/6)	95
*Albumin Vehicle*	100%(3/3)	100%(3/3)	100%(3/3)	100%(3/3)	71

*p* values were calculated using Fisher’s Exact Test between treatment groups (*P ≤ 0.05).

### Survival analysis

**Figure 1:**
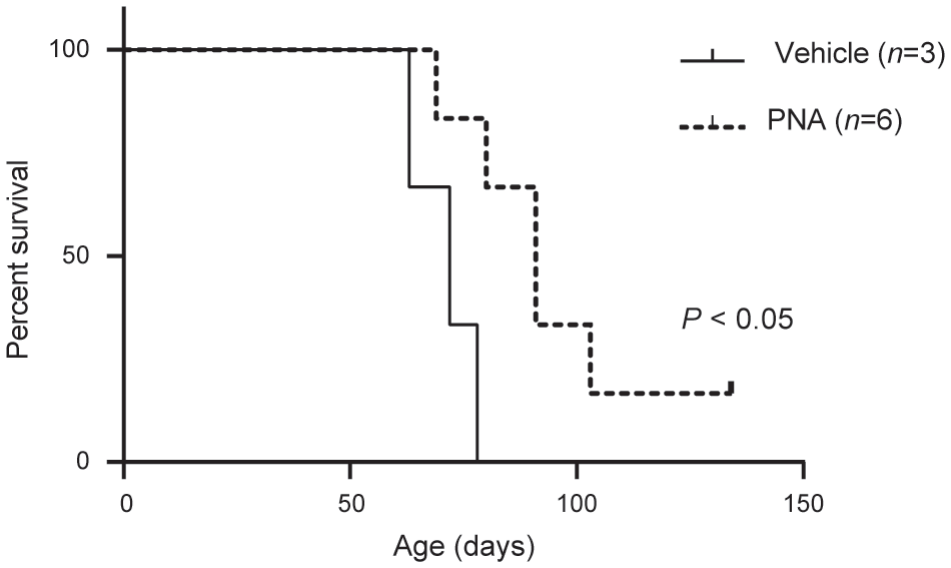
Kaplan-Meier curve. PNA treatment increased the survival of MAP mice.

Kaplan-Meier survival analysis (**Figure 1**) showed MAP mice treated with albumin vehicle had a mean survival of 71 days (n = 3) while PNA treated MAP mice had a mean survival of 95 days (n = 6, *P <*0.05, **Figure 1**). Thus, PNA increased lifespan by ~28%.

### Cell proliferation and differentiation

**Figure 2:**
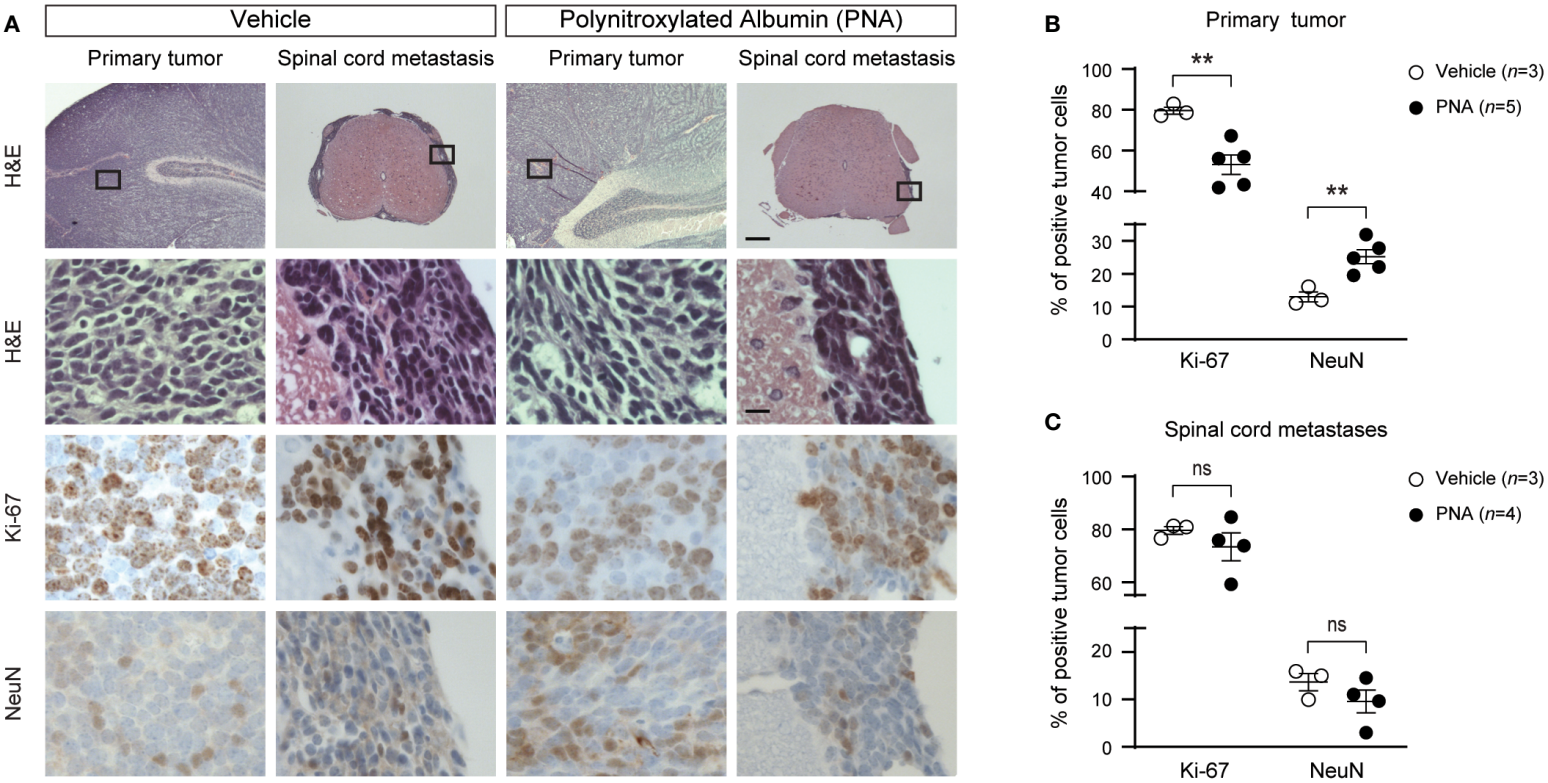
Effect of PNA treatment on proliferation and differentiation of tumors in MAP mice. **(A)** Representative results of hematoxylin and eosin (H&E) staining and immunohistochemical staining for Ki-67^+^ and NeuN^+^ are shown in primary and spinal metastatic tumor from MAP mice treated with PNA or vehicle. Boxed regions in top panel are magnified in lower panels. Scale bars, 250μm (top panel) and 12.5 μm (lower panels). **(B)** Quantitation of the percentage of Ki-67^+^ or NeuN^+^ tumor cells in primary tumor is shown (n = 3 for vehicle, n = 5 for PNA treatment; mean ± s.e.m., two-tailed unpaired t-test, **P<0.01). **(C)** Quantitation of the percentage of Ki-67+ or NeuN^+^ tumor cells in spinal metastatic tumor is shown (n = 3 for vehicle, n = 4 for PNA treatment; mean ± s.e.m., two-tailed unpaired t-test, ns, not significant).

Cell proliferation and differentiation in primary and secondary tumors were evaluated using quantitative Ki-67^+^ and NeuN^+^ immunohistochemistry (**Figure 2**) in animals at end point. Ki-67^+^ and NeuN^+^ are used as markers for mitotic cells and postmitotic cells, respectively (39). Ki-67^+^ expression was significantly reduced by PNA in primary brain tumors (**Figure 2B**, **P<0.01) but not in spinal cord tumors (**Figure 2C**). NeuN^+^ expression as a measure of differentiation increased in primary tumors but was not significantly changed in secondary tumors (**Figures 2B, C**). Thus, PNA treatment significantly affected cell proliferation and differentiation only in primary tumors.

### Spinal cord tumor burden and cell density

**Figure 3:**
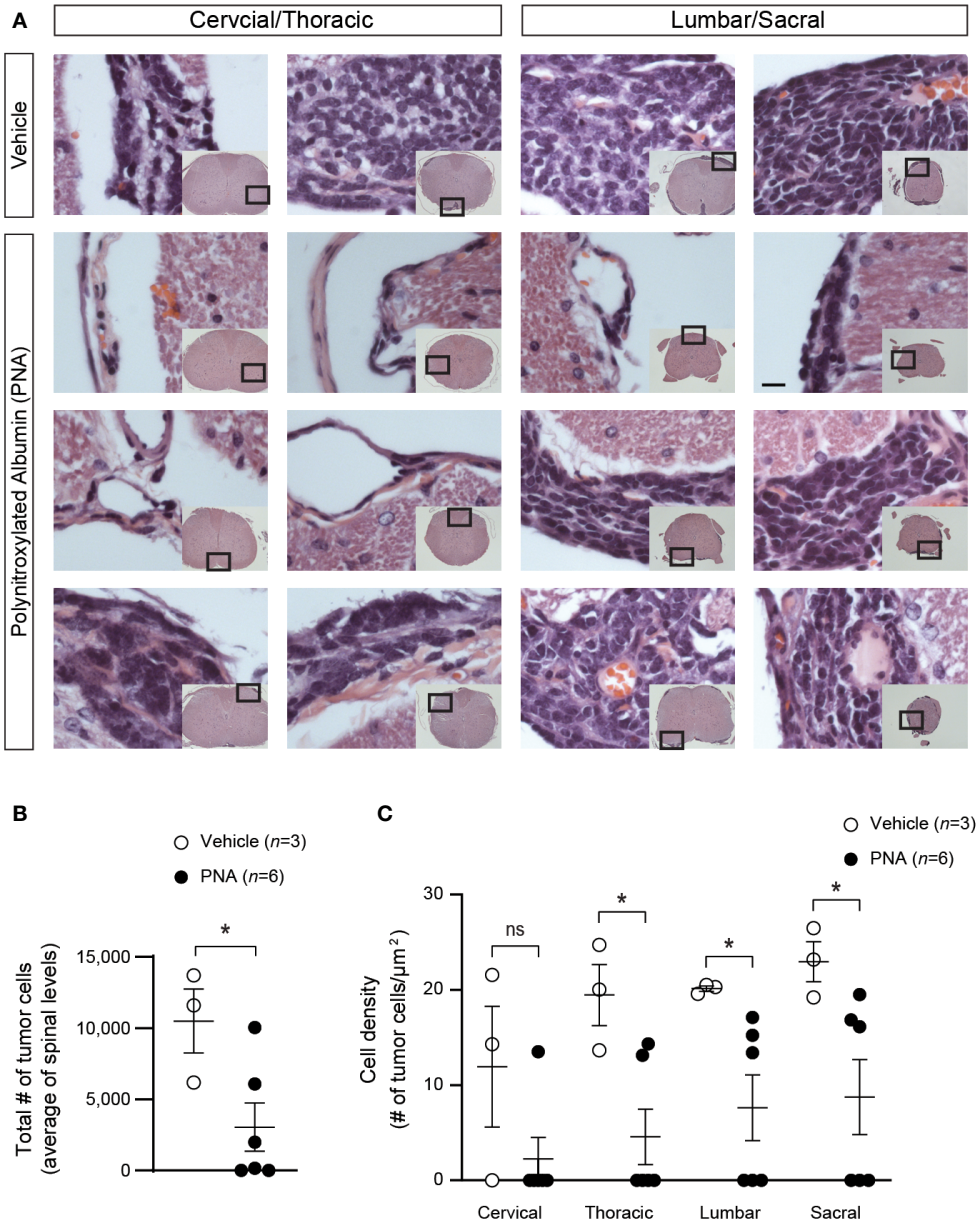
Effect of PNA treatment on spinal cord metastasis in MAP mice. **(A)** Representative results of H&E staining are shown in metastatic tumors at different spinal cord regions in MAP mice treated with PNA or vehicle. Boxed regions of the cross sections of the spinal cords in inset pictures are shown in higher magnification. Scale bar, 12.5 μm. **(B)** Quantitation of spinal metastatic tumor cell numbers is shown in MAP mice treated with PNA or vehicle. (n = 3 for vehicle, n = 6 for PNA treatment; mean ± s.e.m., two-tailed unpaired test *P<0.05). **(C)** Quantitation of metastatic tumor cell density is shown at different levels of the spinal cord (n = 3 for vehicle, n = 6 for PNA treatment; mean ± s.e.m., two-tailed unpaired t-test, *P<0.05; ns, not significant).

The effects of PNA on tumor burden and cell densities in spinal cord was evaluated in animals at endpoint using H&E staining (**Figure 3A**). Tumor burden (mean total number of cells) for the entire spinal cord was significantly reduced in mice treated with PNA compared to albumin vehicle (**Figure 3B**, *P*<0.05). The density of cells (number of cells/µm2) in tumors at four levels of the spinal cord was quantified (**Figure 3C**). MAP mice treated with PNA were found to have significantly reduced metastatic cell density in the thoracic, lumbar and sacral regions (*P*<0.05), while cell density in the cervical region did not show significant change.

## Discussion

Treatment of MAP mice with PNA completely prevented secondary tumor formation in the forebrain in 5/6 (83%) of animals and prevented secondary tumor formation in the spinal cord in 2/6 (33%) of animals, while all untreated animals had both. Concomitantly, lifespan was prolonged by nearly 30%. These effects are especially striking as the Sonic Hedgehog MB subtype is known to not be readily amenable to pharmacological intervention owing to an intact BBB (40, 41). The tumor microenvironment in all MB subtypes is also an immunosuppressive environment that prevents the recruitment and activity of endogenous immune cells (42).

PNA administration was started at 6 weeks of age when primary cerebellar tumors are first seen. However, our transgenic model has enhanced metastasis and small clumps of tumor cells are also already present in the CSF at 6 weeks (39). This protocol mimicked the clinical scenario where treatment is typically initiated only once tumors are present. Had treatment been started earlier, any inhibition of metastasis might result more from reducing tumor burden rather than from an effect on secondary tumor formation, causing false positives. Although the prevention of metastasis was most striking in forebrain brain cortex, due to the near absence of tumor lesions in forebrain we focused on primary and spinal cord tumors in further analyses.

Immunohistochemical analyses showed that PNA significantly reduced the proliferative index and increased differentiation in primary tumors but not in spinal cord tumors. Direct counting of the number of cells in spinal cord tumors, however, showed that the mean total number of cells was significantly reduced in mice treated with PNA. The results suggest that PNA causes a reduction in the ability of cancer cells to establish new tumors at distant sites but the lack of apparent change in proliferation and differentiation in spinal cord is puzzling. This may have been because PNA caused a shift in the balance between proliferation and cell death or PNA affected the tumor environment in a way that did not actually affect differentiation or proliferation.

For spinal cord tumors, we observed a significant decrease in metastatic cell density at the thoracic, lumbar and sacral regions but not in the cervical region. The lack of significant change at the cervical level could be due as one possibility to random variation because the number of mice used was low. Alternatively, there may be differences in the tumor microenvironment at different levels causing tumor cells to respond differently or pharmacokinetic properties may be different in different spinal regions. Different drug levels might result, for example, from differences in local blood flow or differences in the ability to cross the BBB and/or blood-spinal cord barrier.

### Blood brain barrier and the pharmacological distribution of PNA

Numerous studies have shown that PNA is a vascular therapeutic in animal models of stroke, traumatic brain injury, sickle cell disease, myocardial infarction, and preventing lung capillary leak (27, 30–35, 37). The sustained therapeutic effects observed in all indications were thought to be derived from responses within the vasculature. In agreement, PNA’s demonstrated vascular effects to date have included reversal of hypoxia, blood flow enhancement, modulation of NO signaling, and alleviation of oxidative and nitrosative stress. It is unclear whether the effects of PNA on metastasis in MB requires crossing the BBB and entry into the CSF. The BBB is intact in SSH MB while in WNT MB it is leaky (40, 41). As a result, WNT MB is considered more treatable compared to SHH MB (40, 41). However, it is possible that highly local geographic variation in BBB leakiness could exist in SHH MB as suggested in glioma (43), despite overall integrity of the barrier in this MB subtype. Although data on the geographic variability of CNS drug penetration is quite sparse, we cannot rule out loss of BBB integrity in the specific vicinity of tumors in our model while the bulk of the BBB remains intact. On the other hand, given recent work by Taylor and colleagues on hematogenous dissemination of MB (9), it is also possible that PNA exerts its anti-tumor effects in MB without crossing the BBB.

Determining PNA’s concentration in CSF in treated animals would help in addressing whether PNA is acting within the CSF, yet is made difficult by the small volume of CSF in mice as well as the risk of contamination from blood. The distribution of PNA is thought to be similar to the well-studied distribution of serum albumin (44, 45). The BBB and the blood-spinal cord barrier allow only very low levels of albumin in CSF. In contrast, BBB disruption or leakiness associated with pathological conditions leads to increased albumin levels in CSF (2, 44). Entry of albumin into the CNS normally is an active process involving receptor-mediated internalization into endothelial cells and transcytosis dependent on the albumin-binding protein SPARC (45). SPARC has been found to be over-expressed in glioma and provides a mechanism for albumin-based drug delivery to the CNS (46, 47). The expression of SPARC and its potential role in PNA transport into the CSF in SHH MB will need investigation.

Assuming an intact BBB and lack of enhancement in SPARC-mediated transport, the concentration of albumin in CSF is normally ~3 µM (48), a >200 fold lower concentration than in plasma (~640 µM). In the elderly or in chronic pathological conditions where the BBB is thought to be leaky, the CSF albumin concentration increases only ~2 fold (48, 49). Although CSF albumin levels in brain cancers such as MB and glioblastoma have not been directly measured, mass spectrometry data supports slightly increased albumin levels in glioblastoma (50).

In previous *in vitro* studies on DAOY medulloblastoma (21), U-87 glioblastoma cells (21) and in 4T1 breast cancer cells (17), the effective PNA concentration for inhibiting tumor cell proliferation was 30-120 µM. The amount of PNA administered in this study predicts a PNA concentration of ~49 µM in plasma (an ~8% increase in total serum albumin). If PNA distributes similarly to endogenous albumin and the BBB isn’t appreciably leaky, then PNA within the CSF would be ~0.25 µM in our experiments, 100-480 fold lower than the concentrations effective in suppressing tumor cell proliferation (17, 21). Yet, PNA exerted significant effects on MB at primary and metastatic sites in our model. These observations argue that PNA is acting from within the vasculature. PNA’s concentration in CSF would have to rise 100-480 fold to be effective. Although significant increases in CSF albumin levels have been seen to occur transiently in trauma situations (51), BBB disruption to the extent needed to reach effective PNA levels in CSF would have immediate life-threatening consequences and couldn’t be sustained for long. Therefore, our results suggest that tumor suppression from PNA results from PNA’s effects within the vasculature.

### Mechanistic considerations

The anticancer effects of PNA mainly result from the covalently attached caged nitric oxide groups. While serum albumin on its own has anti-oxidative effects (52), total albumin in plasma (PNA + endogenous albumin) in our experiments increased only by ~8% after administration of PNA or control serum albumin (see above). Low molecular weight cyclic nitroxides are known to be superoxide dismutase (SOD)-mimetics, act as antioxidants by oxidizing cuprous and ferrous ions, and also react directly with a variety of free radicals to detoxify them e.g. lipid peroxidation (16). Numerous studies have shown the efficacy of low molecular nitroxides in animal models (13–15, 53). However, as they easily cross membranes, they are toxic and subject to rapid reduction intracellularly to the inactive hydroxylamine form. Covalent attachment of CNO groups to a macromolecule, as in PNA, addresses these issues by localizing activity extracellularly, prolonging pharmacological effects and preventing toxicity.

Imbalances in antioxidant defenses are one of the hallmarks of cancer (13, 15). There are 3 superoxide dismutases, two of which are intracellular (SOD1 and SOD3) and one is extracellular (SOD3/EcSOD) (15). Low molecular weight cyclic nitroxides on their own can act as SOD mimetics of all three enzymes (13, 15), but when coupled to PNA no longer cross membranes and can only act as an SOD3 mimetic in the vasculature. Without PNA, nitric oxide (NO) reaction with elevated superoxide in the vasculature leads to depletion of NO levels and production of peroxynitrite. Peroxynitrite is where the oxidative and nitrosative stress pathways merge, and peroxynitrite is the major free radical in the pathogenesis of multiple diseases. Lowering of vascular superoxide with PNA would reduce peoxynitrite production and oxidative/nitrosative stress.

Hypoxia is well known in solid tumors and has been shown in glioblastoma to limit the effectiveness of chemotherapeutics (54). The CSF in MB is a hypoxic environment (55) while a derangement in ROS homeostasis promotes metastasis of SHH MB through stabilization of hypoxia-inducible factor 1α (HIF1α) (56). It has been shown using live animal imaging that PNA can increase blood flow within minutes in peripheral solid tumors (17, 18). Thus, in addition to reducing oxidative/nitrosative stress, the lowering of superoxide and elevation of NO levels with PNA would also increase blood flow to tumors, resulting in decreased hypoxia within tumors.

Anti-hypoxic effects are also seen with low molecular weight cyclic nitroxides that can cross membranes and act intracellularly. TEMPOL is one such example and has been shown to inhibit both hypoxia-inducible factor 1α (HIF-1α) and hypoxia-inducible factor 2 α(HIF-2α) in glioblastoma (54). Compared with PNA, these nitroxides do not show the vascular and therapeutic effects observed with PNA in diverse animal disease models. Nevertheless, comparison of the effects of these two classes of drugs in inhibiting dissemination and metastasis is warranted.

If hematogenous dissemination occurs in our transgenic model, the main candidate locations where PNA may be acting are:

1. The tumor microenvironment (TME) of primary cerebellar tumors.
2. Translocation of circulating tumor cells (CTCs) from CSF into the vasculature.
3. Translocation of CTCs from the vasculature into CSF.
4. The TME of secondary tumors

The TME of brain tumors is comprised of diverse cell types including malignant cells, astrocytes, macrophages/microglia, neurons, pericytes and endothelial cells (57–59). PNA may affect any of these cells as well as the dynamically changing extracellular matrix. The glycocolyx on the luminal site of endothelial cells may also be important owing to the central role of albumin in its properties (60). For peripheral solid tumors, it has been suggested that the vasculature can also adopt a hybrid structure that incorporates primary tumor cells (61), which if operative in MB would make tumor cells directly accessible to PNA from the luminal side of the vasculature.

The CCL2 pathway receptor is known to play an important role in the transit of leukocyte populations ito the CNS (62, 63). Recently, the CCL2 pathway has also been implicated to be involved in the reentry of CTCs into CSF in MB (9). In this respect, PNA might affect the CCL2 pathway in the entry of CTCs into the CSF.

Finally, all MB subtypes are characterized as having an immunosuppressive tumor environment and are considered not amenable to immunotherapy (42). As elevated NO levels after PNA treatment may help to create a favorable tumor environment for endogenous immune cell functions, this mechanism would not require the involvement of hematogenous dissemination of CTCs.

## Conclusions

This is the first report to show that PNA, a representative of a new class of drugs referred to as caged nitric oxide nanoparticles, greatly inhibits metastasis in cancer. Inhibition of leptomeningeal dissemination and metastasis in MB is demonstrated. This finding extends a previous study in a triple negative breast cancer murine model where PNA was found to inhibit metastasis only when co-administered with standard of care chemotherapeutics and not if administered on its own (17). PNA is ideally suited for use as a conjunctive agent to be used in conjunction with other types of cancer therapies including chemotherapy, radiotherapy, surgery and immunotherapy. Future study of other MB subtypes, of xenograph models and of other brain cancers is warranted in order to further delineate the relevance of our findings to humans. Modulation of the immunosuppressive environment of tumors by PNA is also an exciting possibility.

## Data availability statement

The original contributions presented in the study are included in the article/supplementary material. Further inquiries can be directed to the corresponding authors.

## Ethics statement

The animal study was reviewed and approved by Sanford Research Institutional Animal Care and Use Committee (IACUC).

## Author contributions

KG, BS, CH, and HZ conceptualized different aspects of the study. KG performed the experiments, analyzed results and prepared figures. BS analyzed data and wrote the manuscript. SM and CH reviewed results and the manuscript. HZ oversaw all research, performed data analysis and approved the final manuscript. All authors contributed to the article and approved the submitted version.
